# Impact of thyroid dysfunction and diabetes on fibrosis and steatosis stages in metabolic dysfunction associated fatty liver disease

**DOI:** 10.1038/s41598-025-28232-x

**Published:** 2025-12-15

**Authors:** Mohamed W. Saleh, Maysa I. Farghly, Eman Elsebaie, Nessren Mohamed, Eman El-Mankhly

**Affiliations:** 1 Internal Medicine Department, Faculty of Medicine, Suez University, Suez, Egypt; 2 Clinical Pathology Department, Faculty of Medicine, Suez University, Suez, Egypt; 3 Public health and community Medicine Department, Faculty of Medicine, Cairo University, Cairo, Egypt; 4 Hepatogastroentrology and Infectious Diseases Department, Faculty of Medicine, AL-Azhar University, Cairo, Egypt; 5 Internal Medicine Department, Faculty of Medicine, Al-Azhar University, Cairo, Egypt

**Keywords:** Non-alcoholic fatty liver disease, Steatosis, Diabetes mellitus, Primary hypothyroidism, FibroScan, Endocrinology, Gastroenterology

## Abstract

Metabolic dysfunction associated fatty liver disease (MAFLD) is a common metabolic disorder intricately linked to diabetes and thyroid dysfunction. Hypothyroidism, characterized by elevated thyroid-stimulating hormone (TSH), may disrupt lipid metabolism and exacerbate insulin resistance, contributing to MAFLD progression. Investigating the combined impact of diabetes and thyroid dysfunction on fibrosis and steatosis stages in MAFLD is essential for optimizing disease management. This prospective cross-sectional study was conducted at Suez General Hospital from March to September 2024 and included 400 patients with MAFLD **.** Participants were stratified into four groups: Group A: 100 diabetic patients without thyroid dysfunction; Group B: 100 non-diabetic patients without thyroid dysfunction; Group C: 100 diabetic patients with thyroid dysfunction; and Group D: 100 non-diabetic patients with thyroid dysfunction. Clinical assessment, laboratory investigations, fibrosis-4 (FIB-4) scores, and abdominal ultrasonography were performed for all participants, and transient elastography (FibroScan^®^) for those with FIB-4 scores > 3.25. Significant differences were observed in the distribution of body mass index (BMI), liver enzymes, and fibrosis stages across the groups (*P* < 0.001, for all). Group C exhibited the highest prevalence of advanced fibrosis (F2–F3), while severe steatosis (S3) was predominant in Group D. Hypothyroidism and subclinical hypothyroidism were associated with elevated FIB-4 scores and advanced steatosis (*p* = 0.033), highlighting the impact of thyroid dysfunction on MAFLD progression. Diabetes and thyroid dysfunction can exacerbate MAFLD severity, emphasizing the need for integrated management strategies targeting these comorbidities to mitigate fibrosis and steatosis progression.

## Introduction

Metabolic dysfunction-associated fatty liver disease (MAFLD) and thyroid disease are highly prevalent globally and are reported to be present as comorbid conditions in up to 22.4% of patients with MAFLD. Primary hypothyroidism, henceforth referred to as hypothyroidism, is a disease characterized by a TSH concentration above the reference range; it can either be overt or subclinical^1^.

Metabolic dysfunction-associated fatty liver disease (MAFLD) is the most frequent cause of chronic liver disease that affects millions of people worldwide. Genetics, obesity, unhealthy lifestyle, and other metabolic risk factors could be responsible for the burgeoning evolution, increased prevalence, and incidence of MAFLD^2^.

MAFLD encompasses a wide range of liver conditions from simple steatosis/ metabolic dysfunction-associated fatty liver disease (MAFLD) to MASH (Metabolic Dysfunction-Associated Steatohepatitis), cirrhosis, and finally, hepatocellular carcinoma (HCC)^3^. Considering the common and possible evolution to MASH and cirrhosis, MAFLD has become one of the main causes of liver transplantation^4^.

Hypothyroidism continues to remain a global problem with increased incidence among the adult and newborn population, and illustrates decreased metabolic rate determined by hypo secretion of TSH from the thyroid gland^5^. Studies have shown that patients with over ten years of thyroid dysfunction have significantly higher chances of developing HCC^6^, and that in subjects with MASH and chronic hepatitis B infection, a higher thyroid dysfunction was found compared to that of the control group^7^. The hypothalamic pituitary thyroid axis plays an essential role in many metabolic pathways, especially those involving lipids and carbohydrates. MAFLD has been described as the hepatic manifestation of metabolic syndrome. Therefore, a relationship between hypothyroidism and MAFLD has long been hypothesized and studied^8^.

Because of its importance in lipid and carbohydrate metabolism, the involvement of thyroid dysfunction in the pathogenesis of MAFLD seems plausible. Many mechanisms have been implicated in this relationship^9^.

Hyperthyroidism seems to have a protective effect on the incidence of MAFLD. A retrospective case-control study included adult patients (≥ 18 years) with an initial diagnosis of NAFLD in 1262 general practices in Germany found that hyperthyroidism had a protective effect on the risk of incident MAFLD^10^.

### Aim of the work

The study aims to explore the potential role of thyroid dysfunction in Non-Alcoholic Fatty Liver Disease pathogenesis as a risk factor for each other.

## Subjects and methods

### Subjects

The study was carried out in Suez general hospital from march 2024 to September 2024. included 400 patients with thyroid dysfunction divided as follows:

Sample size calculation was based on mean difference of % connection between thyroid dysfunction and nonalcoholic fatty liver disease as a risk factor for each other retrieved from previous research. Using G power program version 3.1.9.7 to calculate sample size based on expected difference of 32.6%, using 2-tailed test, α error = 0.05 and power = 80.0%, the total calculated sample size will be 50 in each group at least.

#### Group A

will include 100 DM patients (50 male and 50 female) without thyroid dysfunction and with fatty liver. This group will be divided into 2 subgroups according to FIB-4scor less than 1.45 or more than 3.25.

#### Group B

include 100 non DM patients (50 male and 50 female) without thyroid dysfunction and with fatty liver.

#### Group C

include 100 DM patients (50 male and 50 female) with thyroid dysfunction and fatty liver.

#### Group D

include 100 non DM patients (50 male and 50 female) with thyroid dysfunction and fatty liver.

**Methodology**:

- This study adhered to all relevant ethical guidelines and regulations. All experimental protocols were approved by the Institutional Review Board of the Faculty of Medicine, Suez University. Informed consent was obtained from all participants.

1. **Full history taking**: from all patients and include
  - Age, sex, smoking, consumption of alcohol and coffee and drug history.
  - History of diabetes mellitus (including duration, symptoms, complications, medications such as oral hypoglycemic drugs and insulin).
  - History of other medical conditions as hypertension, liver, autoimmune disease and renal disease.
2. **Clinical examinations**: for all patient with emphasis on vital signs, waist circumference, body mass index (BMI) (kg/m^2^) and abdominal examination.
3. **Investigations**:

**The following laboratory investigations were done;**

1. Routine laboratory investigations including:
  CBC. Hemoglobin concentration was measured with an ADVIA 120 automated hematology analyzer (Bayer, NY, USA).”
  ESR.
  Liver enzymes (ALT, AST) and kidney function (urea, creatinine).
  Complete lipid profile.

Venous blood samples (10 mL) were obtained from subjects after an overnight fast (≥ 12 h). Blood was allowed to clot at room temperature for 15 min and subsequently centrifuged at 3500 rpm for 3 min. Total cholesterol, LDL cholesterol, HDL cholesterol, triglyceride, ALT, AST, blood urea and creatinine levels were determined enzymatically using an automated chemistry analyzer (Toshiba TBA-120 FR, Toshiba Medical Systems, Tokyo, Japan).

2. Glycemic profile including:
  Fasting blood glucose after 8 h fasting, Three milliliters of venous blood were collected in a plain tube and allowed to clot at room temperature for 15 min. Subsequently, the samples were centrifuged at 3500 rpm for 3 min rpm then glucose level was measured in the separated serum using an automated chemistry analyzer (Toshiba TBA-120 FR, Toshiba Medical Systems, Tokyo, Japan).
  Glycosylated hemoglobin (HbA1c). EDTA blood sample for HbA1c using high-performance liquid chromatography method for hemoglobin A1c.

### Principle of HbA1c analyses

HbA1c levels were determined by cation-exchange chromatography after hemolysis and removal of interfering substances. The percentage of HbA1c was calculated by comparing the eluted HbA1c absorbance at 415 nm to the total hemoglobin absorbance.

### Assay process

A. **Preparation of hemolysed solution**:

Fifty µl of blood was pipetted into test tube and then 450 µ of potassium biphthalate was added, mixed gently and incubated at18 22 C^0^ for 10–15 min.

B. **Chromatographic separation** :
  1. Fifty µl of hemolysed solution was added to the column and after 3 min any eventual eluate was discarded.
  2. Two ml buffer was pipetted into the column and the eluate was discarded.
  3. The column was positioned over a test tube, and 2 mL of buffer was added to elute the HbA1c fraction.
  4. The obtained eluate was mixed and the HbA1c fraction absorbance was read at 415 nm against distilled water (AHbA1c).
  5. Preparation of total Hb:

One Handred µ of hemolysed solution was pipetted into a test tube and 12 ml distilled water was added, mixed and the total Hb (A Hb total) absorbance was read at 415 nm using distilled water as a blank.

Calculation: (AHbA1c) / (3×A Hb total) × 100 = %HbA1c.

(4) **FIB-4 score** was calculated for all patients.
(5) **Abdominal ultrasonography and fibro-scan**: Abdominal ultrasonography was done for all patients in the study and fibro-scan was done for patients with FIB-4scor more than 3.25.

## Results

The study was conducted in Suez General Hospital from March 2024 to September 2024 and included 400 patients with fatty liver disease, The study population consisted of 200 females (50%) and 200 males (50%) with a median age of 40 (18–59) years. Half of the participants had diabetes mellitus, while the other half did not. About (46.3%) had thyroid dysfunction, and 3.8% had subclinical hypothyroidism. The majority of patients (88%) had hypertension. The distribution of body mass index (BMI) categories was as follows: 28% were underweight, 37.7% were normal weight, 23.3% were overweight, and 11% were obese. All patients had evidence of fatty liver on ultrasound. Regarding glycemic control, 50% of diabetic patients had normal HbA1c levels, 28% were on target, and 22% had elevated levels. A significant proportion of patients had elevated triglycerides (75%) and cholesterol levels (75%). The distribution of FIB-4 scores was as follows: 30.5% low risk, 47.8% intermediate risk, and 21.8% high risk. Fibro-scan revealed varying degrees of steatosis and fibrosis, with S2 being the most common stage of steatosis (67.8%) and F2 being the most common stage of fibrosis (46.1%) (Table 1). The mean AST level was 42 ± 5 U/L, the mean ALT level was 33 ± 8 U/L. The mean platelet count was 190 ± 95 × 10^9/L. The mean hemoglobin level was 13 ± 2 g/dL (Table 2).

**Table 1:** The demographic characteristics of the study participants:

		Count	%
Gender	Female	200	50.0
	Male	200	50.0
DM	No	200	50.0
	Yes	200	50.0
HTN	No	352	88.0
	Yes	48	12.0
Hypothyroidism	No	215	53.8
	Yes	185	46.3
Sub clinical hypo	No	385	96.3
	Yes	15	3.8
BMI	BMI < 20	127	31.8
	BMI 20–25	147	36.8
	BMI 25–29	86	21.5
	BMI > 30	40	10.0
Ultrasound	Normal	0	0.0
	Fatty liver	400	100.0
HBA1C	Normal	200	50.0
	On target	112	28.0
	Elevated	88	22.0
T.G	Normal	100	25.0
	High	300	75.0
Cholesterol	Normal	100	25.0
	High	300	75.0
FIB-4 score	Low	122	30.5
	Intermediate	191	47.8
	High risk	87	21.8
Fibroscan steatosis %	S0	0	0.0
	S1	11	12.2
	S2	61	67.8
	S3	18	20.0
Fibroscan fibrosis	F1	30	29.4
	F2	47	46.1
	F3	24	23.5
	F4	1	1.0

*DM* diabetes mellitus, *HTN* hypertension, *BMI* body mass index, *T.G* triglycerides, *FIB-4 score* Fibrosis-4 (FIB-4) Index for Liver Fibrosis.

**Table 2:** Laboratory parameters of the study participants:

	Mean	Median	Minimum	Maximum	Standard deviation	Percentile 25	Percentile 75
AST	42	42	35	51	5	39	48
ALT	33	35	20	42	8	27	42
PLT	190	150	96	420	95	126	249
HB	13	14	10	16	2	12	14

*AST* aspartate transaminase, *ALT* alanine transaminase, *PLT* platelet, *HB* hemoglobin.

### Comparison between the four groups regarding different variables

#### Metabolic dysregulation and glycemic profiles

Ultrasound findings confirmed a 100% prevalence of fatty liver among the study population, aligning with the diagnosis of (MAFLD). Laboratory assessments revealed that 75% of patients had elevated triglyceride and cholesterol levels, indicating a high prevalence of dyslipidemia. Among diabetic patients, glycemic control was variable, with 50% having normal HbA1c levels, 28% achieving target levels, and 22% showing elevated HbA1c levels. A statistically significant difference in diabetes prevalence was observed between the study groups (*P* < 0.001). Groups A and C (diabetic patients) exhibited significantly higher proportions of elevated HbA1c levels compared to Groups B and D (non-diabetic patients), highlighting the metabolic burden in diabetic cohorts.

#### Fibrosis and steatosis

Significant differences were observed in the distribution of fibrosis and steatosis stages between the four groups. Group C had a higher proportion of patients with advanced fibrosis (F2 and F3) compared to the other groups. Group D had a higher proportion of patients with severe steatosis (S3) compared to the other groups.

#### Body mass index

A significant difference was observed in the distribution of body mass index (BMI) categories between the four groups (*P* value < 0.001). Group B had a higher proportion of patients with BMI < 20 and BMI 20–25 compared to the other groups.

#### Hypertension

No significant difference was observed in the prevalence of hypertension between the four groups (*P* value = 0.723) (Table 3).

**Table 3:** Comparison between the 4 groups regarding the enlisted variables:

		Group								*P* value
		Group A		Group B		Group C		Group D		
		N	%	N	%	N	%	N	%	
HTN	No	91	91.0	88	88.0	86	86.0	87	87.0	0.723
	Yes	9	9.0	12	12.0	14	14.0	13	13.0	
BMI	BMI < 20	20	20.0	38	38.0	26	26.0	43	43.0	< 0.001
	BMI 20–25	28	28.0	39	39.0	46	46.0	34	34.0	
	BMI 25–29	36	36.0	16	16.0	18	18.0	16	16.0	
	BMI > 30	16	16.0	7	7.0	10	10.0	7	7.0	
Ultrasound	Normal	0	0.0	0	0.0	0	0.0	0	0.0	
	Fatty liver	100	100.0	100	100.0	100	100.0	100	100.0	
HBA1C	Normal	0	0.0	100	100.0	0	0.0	100	100.0	< 0.001
	On target	56	56.0	0	0.0	56	56.0	0	0.0	
	Elevated	44	44.0	0	0.0	44	44.0	0	0.0	
T.G	Normal	52	52.0	0	0.0	28	28.0	20	20.0	< 0.001
	High	48	48.0	100	100.0	72	72.0	80	80.0	
Cholesterol	Normal	52	52.0	0	0.0	28	28.0	20	20.0	< 0.001
	High	48	48.0	100	100.0	72	72.0	80	80.0	
FIB-4 score	Low	44	44.0	44	44.0	0	0.0	34	34.0	< 0.001
	Intermediate	32	32.0	30	30.0	82	82.0	47	47.0	
	High risk	24	24.0	26	26.0	18	18.0	19	19.0	
Fibro scan steatosis %	S0	0	0.0	0	0.0	0	0.0	0	0.0	0.005
	S1	0	0.0	4	16.0	3	17.6	4	16.7	
	S2	16	66.7	12	48.0	13	76.5	20	83.3	
	S3	8	33.3	9	36.0	1	5.9	0	0.0	
Fibro scan fibrosis	F1	12	33.3	6	24.0	7	41.2	5	20.8	0.351
	F2	12	33.3	14	56.0	7	41.2	14	58.3	
	**F3**	12	33.3	4	16.0	3	17.6	5	20.8	
	**F4**	0	0.0	1	4.0	0	0.0	0	0.0	

*DM* diabetes mellitus, *HTN* hypertension, *BMI* body mass index, *T.G* triglycerides, *FIB-4 score* Fibrosis-4 (FIB-4) Index for Liver Fibrosis.

Age and Laboratory Parameter Comparisons Across Groups.

No significant difference in mean age was observed among the four groups (*P* = 0.918). However, notable variations were identified in several laboratory parameters. Liver function tests revealed significant differences in mean AST and ALT levels between the groups (*P* < 0.001), with Group C (diabetic patients with thyroid dysfunction) exhibiting the highest levels. Platelet counts also varied significantly across the groups (*P* < 0.001), with Group C showing significantly lower mean platelet counts compared to the other groups, indicating potential platelet-related abnormalities in this group. Conversely, no significant difference was observed in mean hemoglobin levels between the groups (*P* = 1.000) (Table 4).

**Table 4:** Comparison between the 4 groups as regards age and laboratory parameters:

		Mean	Std. Deviation	Minimum	Maximum	*P* value
Age	Group A	38.71	13.243	19	59	0.918
	Group B	37.72	13.806	19	59	
	Group C	37.98	16.268	18	59	
	Group D	38.96	13.767	19	58	
AST	Group A	41.66	4.628	35	51	< 0.001
	Group B	41.45	5.008	35	49	
	Group C	44.74	4.986	35	49	
	Group D	41.78	5.541	35	49	
ALT	Group A	35.01	6.300	20	42	< 0.001
	Group B	34.21	6.715	20	42	
	Group C	29.88	8.745	20	42	
	Group D	33.78	7.360	21	42	
PLT	Group A	218.84	106.270	96	420	< 0.001
	Group B	210.19	100.488	96	420	
	Group C	142.77	49.305	96	252	
	Group D	188.83	96.257	96	420	
HB	Group A	13.10	1.636	10	16	1.000
	Group B	13.09	1.646	10	16	
	Group C	13.09	1.646	10	16	
	Group D	13.11	1.620	10	16	

*AST* aspartate transaminase, *ALT* alanine transaminase, *PLT* platelet, *HB* hemoglobin.

By investigating the relation between gender and different image studies, we found the following: FIB-4 Score: No significant difference was observed in the distribution of FIB-4 scores between males and females (*p* = 0.428). Fibro-scan Steatosis: No significant difference was observed in the distribution of steatosis stages between males and females (*p* = 0.664). Fibro-scan Fibrosis: No significant difference was observed in the distribution of fibrosis stages between males and females (*p* = 0.754) (Table 5).

**Table 5:** Relation between (FIB-4 score, fibroscan steatosis %, fibroscan fibrosis) and gender:

		Gender				*P* value
		Female		Male		
		N	%	N	%	
FIB-4 score	Low	57	28.5	65	32.5	0.428
	Intermediate	102	51.0	89	44.5	
	High risk	41	20.5	46	23.0	
Fibroscan steatosis %	S0	0	0.0	0	0.0	0.664
	S1	5	11.6	6	12.8	
	S2	31	72.1	30	63.8	
	S3	7	16.3	11	23.4	
Fibroscan fibrosis	F1	14	32.6	16	27.1	0.754
	F2	20	46.5	27	45.8	
	F3	9	20.9	15	25.4	
	F4	0	0.0	1	1.7	

Regarding the relation between hypothyroidism and different image studies: 1- FIB-4 Score: A significant difference was observed in the distribution of FIB-4 scores between patients with and without hypothyroidism (*p* < 0.001). Patients with hypothyroidism had a higher proportion of intermediate and high-risk FIB-4 scores. 2- Fibro-scan Steatosis: A significant difference was observed in the distribution of steatosis stages between patients with and without hypothyroidism. Patients with hypothyroidism had a higher proportion of advanced steatosis (S2 and S3). 3- Fibro-scan Fibrosis: No significant difference was observed in the distribution of fibrosis stages between patients with and without hypothyroidism (*p* = 0.352) (Table 6).

**Table 6:** Relation between (FIB-4 score, fibroscan steatosis %, fibroscan fibrosis) and hypothyroidism:

		Hypothyroidism				*P* value
		No		Yes		
		N	%	N	%	
FIB-4 score	Low	89	41.4	33	17.8	< 0.001
	Intermediate	74	34.4	117	63.2	
	High risk	52	24.2	35	18.9	0.001
Fibroscan steatosis %	S0	0	0.0	0	0.0	
	S1	4	7.8	7	17.9	
	S2	30	58.8	31	79.5	
	S3	17	33.3	1	2.6	
Fibroscan fibrosis	F1	18	28.6	12	30.8	0.352
	F2	26	41.3	21	53.8	
	F3	18	28.6	6	15.4	
	F4	1	1.6	0	0.0	

Regarding the relation between Subclinical Hypothyroidism and different image studies: 1- FIB-4 Score: A significant difference was observed in the distribution of FIB-4 scores between patients with and without subclinical hypothyroidism (*p* = 0.033). Patients with subclinical hypothyroidism had a higher proportion of intermediate and high-risk FIB-4 scores. 2- Fibro-scan Steatosis: No significant difference was observed in the distribution of steatosis stages between patients with and without subclinical hypothyroidism (*p* = 0.615). However, it’s worth noting that the sample size for patients with subclinical hypothyroidism is small, which may limit the statistical power to detect significant differences. 3- Fibro-scan Fibrosis: A significant difference was observed in the distribution of fibrosis stages between patients with and without subclinical hypothyroidism (*p* = 0.085). Patients with subclinical hypothyroidism had a higher proportion of advanced fibrosis (F2 and F3) (Table 7).

**Table 7:** Relation between (FIB-4 score, fibroscan steatosis %, fibroscan fibrosis) and sub clinical hypothyroidism:

		Sub clinical hypothyroidism				*P* value
		No		Yes		
		N	%	N	%	
FIB-4 score	Low	121	31.4	1	6.7	0.033
	Intermediate	179	46.5	12	80.0	
	High risk	85	22.1	2	13.3	
Fibroscan steatosis %	S0	0	0.0	0	0.0	0.615
	S1	11	12.5	0	0.0	
	S2	59	67.0	2	100.0	
	S3	18	20.5	0	0.0	
Fibroscan fibrosis	F1	30	30.0	0	0.0	0.085
	F2	47	47.0	0	0.0	
	F3	22	22.0	2	100.0	
	F4	1	1.0	0	0.0	

Regarding the relation between BMI and different image studies: 1- FIB-4 Score: A significant difference was observed in the distribution of FIB-4 scores across different BMI categories (*p* < 0.001). As BMI increased, the proportion of patients with higher FIB-4 scores (intermediate and high-risk) also increased. 2- Fibro-scan Steatosis: A significant difference was observed in the distribution of steatosis stages across different BMI categories (*p* = 0.078). As BMI increased, the proportion of patients with advanced steatosis (S2 and S3) also increased. 3- Fibro-scan Fibrosis: A significant difference was observed in the distribution of fibrosis stages across different BMI categories (*p* = 0.179). As BMI increased, the proportion of patients with advanced fibrosis (F2, F3, and F4) also increased (Table 8).

**Table 8:** Relation between (FIB-4 score, fibroscan steatosis %, fibroscan fibrosis) and BMI:

		BMI								*P* value
		BMI < 20		BMI 20–25		BMI 25–29		BMI > 30		
		N	%	N	%	N	%	N	%	
FIB-4 score	Low	61	48.0	28	19.0	23	26.7	10	25.0	< 0.001
	Intermediate	59	46.5	91	61.9	32	37.2	9	22.5	
	High risk	7	5.5	28	19.0	31	36.0	21	52.5	
Fibroscan steatosis %	S0	0	0.0	0	0.0	0	0.0	0	0.0	0.365
	S1	2	18.2	4	14.8	1	3.2	4	19.0	
	S2	8	72.7	16	59.3	22	71.0	15	71.4	
	S3	1	9.1	7	25.9	8	25.8	2	9.5	
Fibroscan fibrosis	F1	7	43.8	11	37.9	8	23.5	4	17.4	0.138
	F2	9	56.3	10	34.5	18	52.9	10	43.5	
	F3	0	0.0	8	27.6	8	23.5	8	34.8	
	F4	0	0.0	0	0.0	0	0.0	1	4.3	

*BMI* body mass index.

## Discussion

Metabolic dysfunction associated fatty liver disease (MAFLD) is the leading cause of chronic liver diseases worldwide and a nomenclature that captures fatty liver disease (FLD) with metabolic dysfunctions^11^.

Thyroid function has been proposed as one of the most important risk factors for its prominent effects on hepatic fatty acid and cholesterol synthesis. Normal thyroid function is essential for maintaining liver metabolism, while thyroid disorders affect the clinical progression of liver disease^12^.

In our study, about (46.3%) had thyroid dysfunction, and 3.8% had subclinical hypothyroidism. Similar to our findings, Fan et al.^13^ study included 14,789 patients were strict-normal thyroid function, among them 29.3% had low-normal thyroid function, and 5.6% had subclinical hypothyroidism.

The distribution of BMI categories was as follows: 28% were underweight, 37.7% were normal weight, 23.3% were overweight, and 11% were obese. All patients had evidence of fatty liver on ultrasound.

Regarding glycemic control, 50% of diabetic patients had normal HbA1c levels, 28% were on target, and 22% had elevated levels. A significant proportion of patients had elevated triglycerides (75%) and cholesterol levels (75%). The distribution of FIB-4 scores was as follows: 30.5% low risk, 47.8% intermediate risk, and 21.8% high risk. Fibro-scan revealed varying degrees of steatosis and fibrosis, with S2 being the most common stage of steatosis (67.8%) and F2 being the most common stage of fibrosis (46.1%).

However, Zambrano-Huailla et al.^14^ found that the distribution of fibrosis stages among the sample population was as follows: F0 (45%), F1 (27%), F2 (8%), F3 (16%) and F4 (4%).

A significant difference was observed in the distribution of BMI categories between the four groups. Group B had a higher proportion of patients with BMI < 20 and BMI 20–25 compared to the other groups.

A significant difference was observed in the prevalence of diabetes mellitus between the four groups. Groups A and C, consisting of diabetic patients, had significantly higher proportions of elevated HbA1c levels and higher triglyceride and cholesterol levels compared to Groups B and D.

In alignment with our data, Zhang and colleagues^15^ estimated the prevalence of metabolic dysfunction-associated fatty liver disease (MAFLD) and noted that the prevalence of MAFLD was significantly higher in patients with T2DM than in subjects without diabetes.

All patients in all groups had evidence of fatty liver on ultrasound. Significant differences were observed in the distribution of fibrosis and steatosis stages between the four groups. Group C had a higher proportion of patients with advanced fibrosis (F2 and F3) compared to the other groups. Group D had a higher proportion of patients with severe steatosis (S3) compared to the other groups.

Significant differences were observed in the mean AST and ALT levels between the four groups; group C had significantly higher mean AST and ALT levels compared to the other groups. Also, significant differences were observed in the mean platelet count between the four groups; group C had significantly lower mean platelet count compared to the other groups.

No significant difference was observed in the distribution of FIB-4 scores, the distribution of steatosis stages, and the distribution of fibrosis stages between males and females.

However, Halaoui et al.^16^ assessed the gender impact on liver status in NAFLD patients younger than 50 years and stated that fibrosis score could predict liver status in males but not in females.

On the contrary, Balakrishnan et al.^17^ noted that the risk of advanced fibrosis (RR, 1.56; 95% CI, 1.36–1.80; I2 = 0) was substantially higher in women in study populations with average ages of 50 years and older; sex differences in MASH and advanced fibrosis were attenuated in younger populations. They concluded that once MAFLD is established, women have a higher risk of advanced fibrosis than men, especially after age 50 years.

A significant difference was observed in the distribution of FIB-4 scores and the distribution of steatosis stages, between patients with and without hypothyroidism; patients with hypothyroidism had a higher proportion of intermediate and high-risk FIB-4 scores and advanced steatosis (S2 and S3), but no significant difference was observed in the distribution of fibrosis stages.

Our findings can be explained by the fact that thyroid hormones regulate many metabolic activities in the liver by promoting the export and oxidation of lipids, as well as de novo lipogenesis. They also control hepatic insulin sensitivity and suppress hepatic gluconeogenesis. Because of its importance in lipid and carbohydrate metabolism, the involvement of thyroid dysfunction in the pathogenesis of NAFLD and MAFLD seems plausible^18^.

Similarly, the findings from a previous review by Lugari et al.^19^ support a significant association between primary hypothyroidism and risk of development and progression of fatty liver disease.

In difference, a previous study by D’Ambrosio et al.^20^ demonstrated that rates of hypothyroidism were similar independently of significant fibrosis (*p* = 0.21) or steatosis (*p* = 0.75), this difference may be due to that investigated the association between hypothyroidism and NAFLD histological features potentially associated with progressive liver disease, while our study focused into MAFLD.

A significant difference was observed in the distribution of FIB-4 scores and the distribution of fibrosis stages between patients with and without subclinical hypothyroidism. Patients with subclinical hypothyroidism had a higher proportion of intermediate and high-risk FIB-4 scores and advanced fibrosis (F2 and F3), but no significant difference was observed in the distribution of steatosis stages.

A previous article by Fan et al.^13^ supported our results as they conducted a multivariable regression model adjusted for age, sex, body mass index, type 2 diabetes, and hypertension showed low-normal thyroid function increased the risk of advanced fibrosis in patients with MAFLD (FIB-4 > 2.67: OR = 1.41, 95% CI 1.02–1.93; NFS > 0.676: OR = 1.72, 95% CI 1.08–2.72).

A significant difference was observed in the distribution of FIB-4 scores across different BMI categories, as BMI increased, the proportion of patients with higher FIB-4 scores (intermediate and high-risk) also increased.

A significant difference was observed in the distribution of steatosis stages across different BMI categories. As BMI increased, the proportion of patients with advanced steatosis (S2 and S3) also increased. A significant difference was observed in the distribution of fibrosis stages across different BMI categories. As BMI increased, the proportion of patients with advanced fibrosis (F2, F3, and F4) also increased.

This results were in agreement with Gopalakrishna et al.^21^ who stated that, compared to patients with BMI < 25 kg/m^2^, the adjusted OR (95% CI) of having a higher fibrosis stage was 1.82 (0.61–5.44), 5.93 (2.05–17.13), and 8.56 (2.51–29.17) for patients with BMI of 25 to < 30, 30 to < 40, and ≥ 40 respectively; so, increasing BMI increases the odds of having a higher fibrosis stage.

Additionally, a meta-analysis by Lu et al.^22^ indicated that, obesity (according to ethnic-specific BMI cut-off points to define obesity) could predict a worse long-term prognosis, but they included NAFLD patients.

## Conclusion

This study highlights the high prevalence and progression of MAFLD in patients with underlying metabolic risk factors. The strong associations observed between diabetes, thyroid dysfunction, and elevated BMI with advanced liver fibrosis and steatosis underscore the need for a comprehensive and integrated approach to patient care. Early identification and management of these metabolic risk factors are crucial to preventing the progression of liver disease and improving long-term patient outcomes.

## Data Availability

The datasets generated during and/or analyzed during the current study are available from the corresponding author upon reasonable request.

## References

[1] Tanase, D. M. et al. Hypothyroidism-induced nonalcoholic fatty liver disease (Hin): Mechanisms and emerging therapeutic options. *Int. J. Mol. Sci.* **21**(16),1–29. MDPI AG. (2020). 10.3390/ijms21165927(10.3390/ijms21165927 PMC7460638 32824723

[2] Janota, B., Szczepańska, E., Adamek, B. & Janczewska, E. Hypothyroidism and non-alcoholic fatty liver disease: A coincidence or a causal relationship? *World J. Hepatol.* **15** (5), 641–648. 10.4254/WJH.V15.I5.641 (2023). 37305371 10.4254/wjh.v15.i5.641 PMC10251274

[3] Vidal-Cevallos, P., Murúa-Beltrán Gall, S., Uribe, M. & Chávez-Tapia, N. C. Understanding the relationship between nonalcoholic fatty liver disease and thyroid disease. *Int. J. Mol. Sci.* **24**(19) 10.3390/ijms241914605( (2023). InMultidisciplinary Digital Publishing Institute (MDPI). 10.3390/ijms241914605 PMC10572395 37834051

[4] Fan, H. et al. Low thyroid function is associated with an increased risk of advanced fibrosis in patients with metabolic dysfunction-associated fatty liver disease. *BMC Gastroenterol.* **23**(1). 10.1186/s12876-022-02612-3(2023). 10.1186/s12876-022-02612-3 PMC9814300 36604612

[5] Almomani, A. et al. Prevalence of hypothyroidism and effect of thyroid hormone replacement therapy in patients with non-alcoholic fatty liver disease: A population-based study. *World J. Hepatol.* **14** (3), 551–558. 10.4254/wjh.v14.i3.551 (2022). 35582287 10.4254/wjh.v14.i3.551 PMC9055192

[6] Lonardo, A. et al. Nonalcoholic fatty liver disease: Evolving paradigms. *World J. Gastroenterol.* **23**(36), 6571–6592. Baishideng Publishing Group Co. (2017). 10.3748/wjg.v23.i36.6571 10.3748/wjg.v23.i36.6571 PMC5643282 29085206

[7] Liu, L. et al. Thyroid-stimulating hormone is associated with nonalcoholic steatohepatitis in patients with chronic hepatitis B. *Medicine* **98** (46), e17945. 10.1097/MD.0000000000017945 (2019). 31725651 10.1097/MD.0000000000017945 PMC6867716

[8] Gor, R. et al. Unraveling the role of hypothyroidism in non-alcoholic fatty liver disease pathogenesis: Correlations, conflicts, and the current stand. *Cureus*10.7759/cureus.14858 (2021). 34104598 10.7759/cureus.14858 PMC8174393

[9] Krashin, E., Piekiełko-Witkowska, A., Ellis, M. & Ashur-Fabian, O. Thyroid hormones and cancer: A comprehensive review of preclinical and clinical studies. *Front. Endocrinol.* **10**(FEB) (2019). 10.3389/fendo.2019.00059 10.3389/fendo.2019.00059 PMC6381772 30814976

[10] Labenz, C., Kostev, K., Armandi, A., Galle, P. R. & Schattenberg, J. M. Impact of thyroid disorders on the incidence of non-alcoholic fatty liver disease in Germany. *United European Gastroenterol. J.* **9**(7), 829–836 (2021). 10.1002/ueg2.12124. Epub 2021 Jul 20. PMID: 34288580; PMCID: PMC8435260.(2021). 10.1002/ueg2.12124 PMC8435260 34288580

[11] Le, M. et al. NAFLD Prevalence: A systematic review and meta-analysis. *Clin. Gastroenterol. Hepatol*. **20**, 2809–2017 (2019). 10.1016/j.cgh.2021.12.002 34890795

[12] Ritter, M. J., Amano, I. & Hollenberg, A. Thyroid hormone signaling and the liver. *Hepatology* **72**, 742–752 (2020). 32343421 10.1002/hep.31296

[13] Fan, H. et al. Low thyroid function is associated with an increased risk of advanced fibrosis in patients with metabolic dysfunction-associated fatty liver disease. *BMC Gastroenterol*. **23**, 30–40 (2023). 10.1186/s12876-022-02612-3 PMC9814300 36604612

[14] Zambrano, R. et al. Diagnostic performance of three non-invasive fibrosis scores (Hepamet, FIB-4, NAFLD fibrosis score) in NAFLD patients from a mixed Latin American population. *Ann Hepatol*. **19**, 622–626 (2020). 10.1016/j.aohep.2020.08.066 32919087

[15] Zhang, X. et al. Correlation between thyroid function, sensitivity to thyroid hormones and metabolic dysfunction-associated fatty liver disease in euthyroid subjects with newly diagnosed type 2 diabetes. *Endocrine* **80**, 366–379 (2023). 2023. 36539681 10.1007/s12020-022-03279-2

[16] Halaoui, A., Ali, A., Habib, S., Kanso, M. & Daniel, F. Mukher,j. Gender differences in liver fibrosis among patients younger than 50 years: A retrospective cohort study. *Clin. Res. Hepatol. Gastroenterol*. **44**, 733–738 (2020). 10.1016/j.clinre.2020.01.001 32169461

[17] Balakrishnan, M. et al. Women have a lower risk of nonalcoholic fatty liver disease but a higher risk of progression vs men: A systematic review and Meta-analysis. *Clin. Gastroenterol. Hepatol.* **19**, 61–71 (2021). 32360810 10.1016/j.cgh.2020.04.067 PMC8796200

[18] Vidal, P., Murúa, G., Uribe, M. & Chávez, N. Understanding the relationship between nonalcoholic fatty liver disease and thyroid disease. *Int. J. Mol. Sci.* **24**, 24–36 (2023). (2023). 10.3390/ijms241914605 PMC10572395 37834051

[19] Lugari, S., Mantovani, A., Nascimbeni, F. & Lonardo, A. Hypothyroidism and nonalcoholic fatty liver disease - a chance association? *Horm. Mol. Biol. Clin. Investig.* **41**, 25–30 (2018). 10.1515/hmbci-2018-0047 30367792

[20] D’Ambrosio, R. et al. The relationship between liver histology and thyroid function tests in patients with non-alcoholic fatty liver disease (NAFLD). *PLoS ONE*. **16**, 14–20 (2021). 10.1371/journal.pone.0249614 PMC8023543 33822817

[21] Gopalakrishna, H., Fashanu, O., Nair, G. & Ravendhran, N. Association between body mass index and liver stiffness measurement using transient elastography in patients with non-alcoholic fatty liver disease in a hepatology clinic: A cross sectional study. *Transl. Gastroenterol. Hepatol*. **8**, 10–20(2023). 10.21037/tgh-22-1 PMC9813648 36704647

[22] Lu, F. B. et al. The relationship between obesity and the severity of non-alcoholic fatty liver disease: Systematic review and meta-analysis. *Expert Rev. Gastroenterol. Hepatol.* **12**, 491–502 (2018). 10.1080/17474124.2018.1460202 29609501

